# Developing a risk prediction model for sudden cardiac death in children with hypertrophic cardiomyopathy

**DOI:** 10.3389/fped.2025.1628585

**Published:** 2025-08-14

**Authors:** Zhen Zhen, Yeqiong Xu, Xi Chen, Jia Na, Yanyan Xiao, Yue Yuan

**Affiliations:** Department of Cardiology, Beijing Children’s Hospital, Capital Medical University, National Centre for Children’s Health, Beijing, China

**Keywords:** children, hypertrophic cardiomyopathy, prognosis, sudden cardiac death, predictive model

## Abstract

**Objective:**

This study aimed to develop a predictive model for sudden cardiac death (SCD) in children with hypertrophic cardiomyopathy (HCM).

**Methods:**

The retrospective study included children diagnosed with HCM who visited Beijing Children's Hospital, Capital Medical University between January 2006 and August 2022. Cox regression analysis was used to identify risk factors for SCD. A nomogram was constructed based on risk factors identified through multivariate analysis.

**Results:**

A total of 184 children (115 boys and 69 girls) were included in the study. The median (IQR) age at the initial diagnosis was 4.54 (0.50–10.25) years. Of these, 141 children were diagnosed with primary HCM, while 43 had secondary HCM. The multivariate analysis showed that age <1 year [hazard ratio (HR), 95% confidence interval (CI): 6.232 (2.858–13.591)], female sex [HR: 2.547 (1.460–4.444)], a family history of HCM [HR: 2.622 (1.468–4.683)], pathological Q-waves [HR: 2.290 (1.285–4.082)], fragmented QRS waves [HR: 3.526 (1.786–6.963)], combined arrhythmias (HR: 2.218 [1.136–4.333]), increased interventricular septal thickness [HR: 1.055 (1.008–1.105)], and increased left ventricular posterior wall thickness [HR: 1.060 (1.026–1.096)] were significantly associated with SCD. The nomogram-based SCD prediction model demonstrated strong discriminatory ability, with areas under the curve (AUC) of 0.887 (95% CI: 0.829–0.945) at 1 year, 0.839 (95% CI: 0.777–0.902) at 2 years, 0.847 (95% CI: 0.782–0.912) at 3 years, 0.855 (95% CI: 0.791–0.919) at 4 years, 0.850 (95% CI: 0.789–0.911) at 5 years, and 0.845 (95% CI: 0.763–0.926) at 10 years. Predicted probabilities closely aligned with observed probabilities, indicating good calibration of the model.

**Conclusion:**

A predictive model for SCD in children with HCM was developed, demonstrating strong internal consistency and reliability. External validation is recommended before clinical implementation.

## Introduction

Hypertrophic cardiomyopathy (HCM) is a primary myocardial disease characterized by left ventricular hypertrophy, which may be asymptomatic or associated with variable presentations, including left ventricular outflow tract obstruction (1), diastolic dysfunction, arrhythmias, and sudden cardiac death (SCD) (2). Children younger than one year of age tend to exhibit poorer outcomes, often due to secondary HCM associated with inborn errors of metabolism or syndromes such as RASopathies (3). For children diagnosed after one year of age or those who survive infancy, outcomes are generally more favorable, paralleling those observed in adults (4), largely due to advances in SCD risk stratification and therapeutic innovations. Nonetheless, childhood-onset HCM remains associated with higher lifetime morbidity and mortality attributable to SCD and heart failure (5).

While most studies have focused on risk stratification and prediction models in adult patients with HCM (6–8), the widely recognized HCM Risk-SCD model by Mahony et al., developed using data from 3,675 adult patients with an average age of 48 years, suggests that patients with a ≥4% five-year risk of SCD may benefit from ICD implantation (7). However, diagnosing and assessing risk in children is more challenging. Advanced tools like cardiovascular magnetic resonance (CMR), the gold standard for evaluating heart function and fibrosis, are difficult to use in infants and young children. Additionally, pediatric patients often show vague or subtle symptoms, making risk stratification more complex than in adults.

Nevertheless, certain laboratory and clinical findings have been identified as prognostic indicators in pediatric HCM. Nicoletta et al. demonstrated that NT-proBNP levels correlated with disease progression and were predictive of ICD implantation in pediatric HCM patients (9). Similarly, Miron et al. proposed the PRIMaCY sudden cardiac death risk prediction model based on 572 eligible HCM patients, the model incorporated unique risk factors for pediatric HCM (including baseline characteristics, clinical symptoms, medical history, echocardiographic findings, etc.), and exhibited a prediction accuracy exceeding 70% (10). However, the efficacy of such models remains controversial. Norrish reported poor correlation between PRIMaCY scores and observed risks (11), while Pierre highlighted its low positive predictive value for predicting SCD in pediatric patients (12). Another famous HCM prediction model in children was the HCM Risk-Kids model (13), which was developed through a retrospective, multicentre, longitudinal cohort study encompassing 1,024 patients with HCM (aged ≤16 years) over a 47-year period, and this model could provide individualized estimates of risk at 5 years using readily obtained clinical risk factors. These findings underscore the limitations of adult-derived models and the pressing need for pediatric-specific risk prediction models.

Nomograms have emerged as a novel visualization tool for elucidating the outputs of machine learning models, gaining popularity in recent years. Despite the development of numerous risk stratification and prediction models, their clinical applicability remains suboptimal. For instance, Dai et al. employed a nomogram to interpret the influence of novel biomarkers on HCM progression (14), while Zheng et al. developed an HCM diagnostic model with a C-index of 0.869, subsequently visualized using a nomogram (15). These studies highlight the utility of nomograms in enhancing the interpretability of machine learning-based predictions.

This study aimed to develop a predictive model for SCD in children with HCM.

## Materials and methods

### Study design and participants

This retrospective study included children diagnosed with HCM between January 2006 and August 2022 at Beijing Children's Hospital, Capital Medical University. The inclusion criteria were: (1) patients younger than 18 years at their first visit. (2) Diagnosis of HCM, defined as left ventricular wall thickness >2 standard deviations above the mean (*z*-score >2) for age, sex, or body size, based on clinical manifestations, imaging, and genetic testing (2). The exclusion criteria were: (1) patients who experienced SCD-equivalent events, had a history of cardiopulmonary resuscitation, possessed an ICD, or underwent surgery at another institution. (2) Patients with alternative causes of ventricular hypertrophy, such as hypertension, aortic valve stenosis, congenital subaortic stenosis, or athletic training-induced hypertrophy. (3) Patients with congenital heart disease. (4) Infants of diabetic mothers, including those with gestational diabetes. (5) Patients with myocardial hypertrophy caused by cardiotoxic drugs or prolonged immunosuppressant use.

The study was approved by the Medical Ethics Committee of Beijing Children's Hospital, Capital Medical University (Ethics Approval Number: [2024]-E-016-R). Informed consent was waived due to the retrospective nature of the study.

### Measurements

Echocardiographic examinations were conducted using a Philips iE33 system (probe frequency: 2.5–8.0 MHz) with patients positioned in the supine or lateral position. Images were obtained from the left ventricular (LV) long and short axes at the levels of the mitral valve, papillary muscles, apex, and apical four- and five-chamber views. Measurements included LV end-systolic and end-diastolic diameters, interventricular septum diastolic thickness (IVSd), and LV posterior wall diastolic thickness (LVPWd). Myocardial echocardiographic characteristics were also assessed. The LV ejection fraction (LVEF) was calculated using M-mode echocardiography and adjusted for body surface area. Left ventricular outflow tract obstruction was defined as a pressure gradient ≥30 mmHg at the LV outflow tract, as measured by continuous-wave Doppler ultrasound under resting conditions.

### Outcomes

The primary outcome of this study was defined as a composite of SCD-related events. SCD events included sudden death occurring unexpectedly within 1 h or during the night with previously stable conditions; successful cardiopulmonary resuscitation after cardiac arrest; appropriate implantable cardioverter defibrillator (ICD) discharge for ventricular fibrillation or sustained rapid ventricular tachycardia (>200 beats per min); or sustained rapid ventricular tachycardia or ventricular fibrillation affecting hemodynamic stability and requiring extracorporeal direct current cardioversion.

### Data collection

The clinical characteristics of patients, including age, sex, etiology, family history, clinical manifestations, and NYHA heart failure classification at initial diagnosis (3, 4), as well as supplementary examination results (including chest radiography, electrocardiography [ECG], echocardiography, and enhanced CMR for late gadolinium enhancement [LGE] evaluation), were extracted from electronic medical records.

A positive family history was defined by the presence of one or more of the following conditions: (1) a confirmed diagnosis of HCM in primary or secondary relatives; (2) sudden death of a first- or second-degree relative before the age of 40; (3) a history of unexplained syncope in first- or second-degree relatives.

### Statistical analysis

Statistical analyses were performed using R software (version 3.6.1) and SPSS 26.0 (IBM Corp., Armonk, N.Y., USA). Categorical data were expressed as counts and percentages (*n* [%]) and compared using the chi-square test or Fisher's exact test. Normally distributed continuous data were expressed as mean ± standard deviation (SD) and compared using the Student's *t*-test. Skew distributed continuous data were presented as medians and interquartile ranges (IQR) and compared using the Mann–Whitney *U* test. Variables demonstrating statistical significance in the univariate cox regression analysis were included in the multivariate cox regression analysis and selected using the stepwise method.

A predictive model was constructed using 10-fold cross-validation. Internal validation of the nomogram was performed with a 2,000-iteration bootstrap method. Model performance metrics, including the area under the receiver operating characteristic curve (AUC), accuracy, sensitivity, specificity, positive predictive value (PPV), and negative predictive value (NPV), were calculated. A two-tailed *P*-value <0.05 was considered statistically significant.

## Results

### Patient characteristics

This study included 184 children (62.5% boys) with HCM. The median follow-up duration was 48 months (IQR: 14.70–88.38 months). During the follow-up period, 56 children (30.4%) reached the study's composite endpoint, comprising 36 cases of SCD, 18 instances of successful resuscitation from cardiac arrest, and 2 cases of ventricular tachycardia terminated by electrical cardioversion or ICD discharge. Children in the SCD group showed distinct differences compared to the non-SCD group. They were more likely to be under 1 year old (44.64% vs. 28.91%, *P* = 0.038), with more females (50.00% vs. 32.03%, *P* = 0.021), secondary etiologies (37.50% vs. 17.19%, *P* = 0.003), and family history (41.07% vs. 20.31%, *P* = 0.003). Symptoms like fatigue, syncope, and NYHA III–IV status were more frequent, as were enlarged cardiac silhouette, ST-T changes, and arrhythmias, including SVT and WPW syndrome. They also had higher IVSd, LVPWd, and reduced LVEF, although only 51.6% (*n* = 95) of all patients underwent enhanced CMR examination, we still noticed that LGE positive rate was significantly higher in SCD group compared to non SCD group (55.17% vs. 22.73%, *P* = 0.002), highlighting more severe structural and functional abnormalities (Supplementary Table S1).

Twenty-eight children were lost to follow-up. Seven children underwent modified Morrow surgery to excise hypertrophic myocardium 3–5 mm proximal to the anterior mitral junction. Two children underwent heart transplantation and ICD implantation.

### Risk factors for prognosis of HCM in children

Because 48.4% of children (*n* = 89) did not undergo CMR examination, LGE positivity was excluded from further analysis. Multivariate analysis revealed that age <1 year (HR: 6.232; 95% CI: 2.858–13.591; *P* < 0.001), female sex (HR: 2.547; 95% CI: 1.460–4.444; *P* = 0.001), family history (HR: 2.622; 95% CI: 1.468–4.683; *P* = 0.001), pathological Q waves (HR: 2.290; 95% CI: 1.285–4.082; *P* = 0.005), fQRS (HR: 3.526; 95% CI: 1.786–6.963; *P* < 0.001), arrhythmia (HR: 2.218; 95% CI: 1.136–4.333; *P* = 0.020), increased IVSd (HR: 1.055; 95% CI: 1.008–1.105; *P* = 0.022), and increased LVPWd (HR: 1.060; 95% CI: 1.026–1.096; *P* = 0.001) were independent risk factors for SCD in children with HCM (Table 1).

**Table 1:** Factors influencing the prognosis of HCM in children.

Variable	Univariate		Multivariate	
	HR (95%CI)	*P*	HR (95%CI)	*P*
Age				
<1 year	1.710 (1.008, 2.900)	0.046	6.232 (2.858, 13.591)	<0.001
≥1 year	1.0 (reference)		1.0 (reference)	
Sex				
Female	1.947 (1.151, 3.293)	0.013	2.547 (1.460, 4.444)	0.001
Male	1.0 (reference)		1.0 (reference)	
Etiology				
Secondary	2.511 (1.459, 4.322)	0.001		
Primary	1.0 (reference)			
Family History	2.278 (1.336, 3.883)	0.002	2.622 (1.468, 4.683)	0.001
Initial symptoms				
Chest pain/tightness	0.584 (0.276, 1.235)	0.159		
Fatigue/decreased exercise tolerance	2.110 (1.232, 3.613)	0.007		
Palpitations	0.485 (0.118, 1.990)	0.315		
Syncope	2.196 (1.159, 4.162)	0.016		
Cardiogenic shock	2.508 (1.185, 5.304)	0.016		
Atypical presentation	1.598 (0.685, 3.732)	0.278		
Initial signs				
Murmur	0.875 (0.502, 1.523)	0.636		
Gallop rhythm	1.272 (0.176, 9.197)	0.812		
Initial heart failure classification				
Class III–IV	2.776 (1.626, 4.739)	<0.001		
Chest x-ray				
Enlarged cardiac silhouette	2.950 (1.669, 5.216)	<0.001		
Pulmonary congestion	3.034 (0.947, 9.723)	0.062		
Electrocardiogram (ECG)				
ST-T changes	3.009 (1.088, 8.323)	0.034		
Pathological Q waves	3.242 (1.902, 5.524)	<0.001	2.290 (1.285, 4.082)	0.005
Fragmented QRS	2.630 (1.554, 4.452)	<0.001	3.526 (1.786, 6.963)	<0.001
Left ventricular hypertrophy	1.881 (0.971, 3.647)	0.061		
NSVT (nonsustained ventricular tachycardia)	1.681 (0.959, 2.947)	0.070		
Supraventricular tachycardia (SVT)	1.723 (1.010, 2.937)	0.046		
Atrioventricular (AV) block	1.217 (0.664, 2.229)	0.525		
Wolff-Parkinson-White (WPW) syndrome	2.637 (1.414, 4.916)	0.002		
Arrhythmia	2.546 (1.389, 4.665)	0.003	2.218 (1.136, 4.333)	0.020
Echocardiography				
LVDd, (mm)	0.998 (0.969, 1.027)	0.892		
LVDs, (mm)	0.988 (0.956, 1.020)	0.456		
IVSd, (mm)	1.035 (0.999, 1.071)	0.057	1.055 (1.008, 1.105)	0.022
LVPWd, (mm)	1.057 (1.030, 1.086)	<0.001	1.060 (1.026, 1.096)	0.001
IVSd/LVPWd	0.885 (0.663, 1.181)	0.407		
LVEF, (%)	0.972 (0.955, 0.989)	0.002		
LVOTO	0.840 (0.424, 1.666)	0.619		
CMR#				
LGE positive	2.716 (1.299, 5.677)	0.008		

LGE was excluded from the multivariate analysis as 89 individuals did not undergo a CMR examination. CMR, cardiovascular magnetic resonance; LVDd, LV end-diastolic diameter; LVDs, LV end-systolic diameter; IVSd, interventricular septum diastolic thickness; LVPWd, LV posterior wall diastolic thickness; LVEF, LV ejection fraction; LVOTO, LV outflow tract obstruction; LGE, late gadolinium enhancement.

### Predictive model for SCD in children with HCM

A predictive model for SCD in pediatric HCM patients was developed based on the results of the multivariate analysis (Supplementary Figure S1). The model demonstrated robust predictive performance, with AUC values of 0.887 (95% CI: 0.829–0.945) at 1 year, 0.839 (95% CI: 0.777–0.902) at 2 years, 0.847 (95% CI: 0.782–0.912) at 3 years, 0.855 (95% CI: 0.791–0.919) at 4 years, 0.850 (95% CI: 0.789–0.911) at 5 years, and 0.845 (95% CI: 0.763–0.926) at 10 years (Supplementary Figure S2). Bootstrap confidence intervals were sufficiently narrow, ensuring the stability of the model predictions (Table 2).

**Table 2:** Results of the predictive performance of the risk prediction model.

Time	AUC	95% CI	*P*
1-year	0.887	0.829, 0.945	<0.001
2-year	0.839	0.777, 0.902	<0.001
3-year	0.847	0.782, 0.912	<0.001
4-year	0.855	0.791, 0.919	<0.001
5-year	0.850	0.789, 0.911	<0.001
10-year	0.845	0.763, 0.926	<0.001

### Development and validation of a nomogram for predicting the prognosis of HCM in children

**Figure 1:**
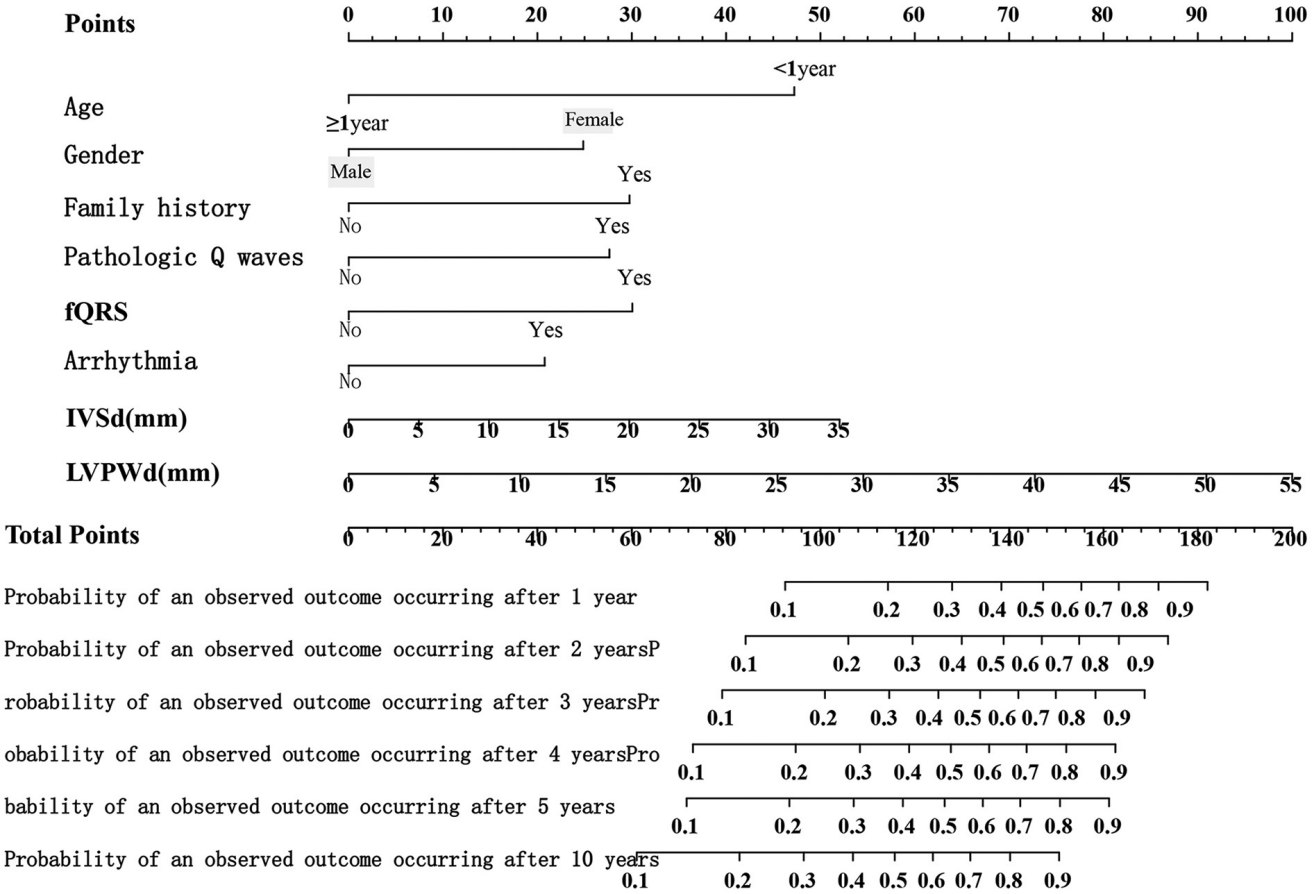
Nomogram of the risk prediction model for SCD in children.

**Figure 2:**
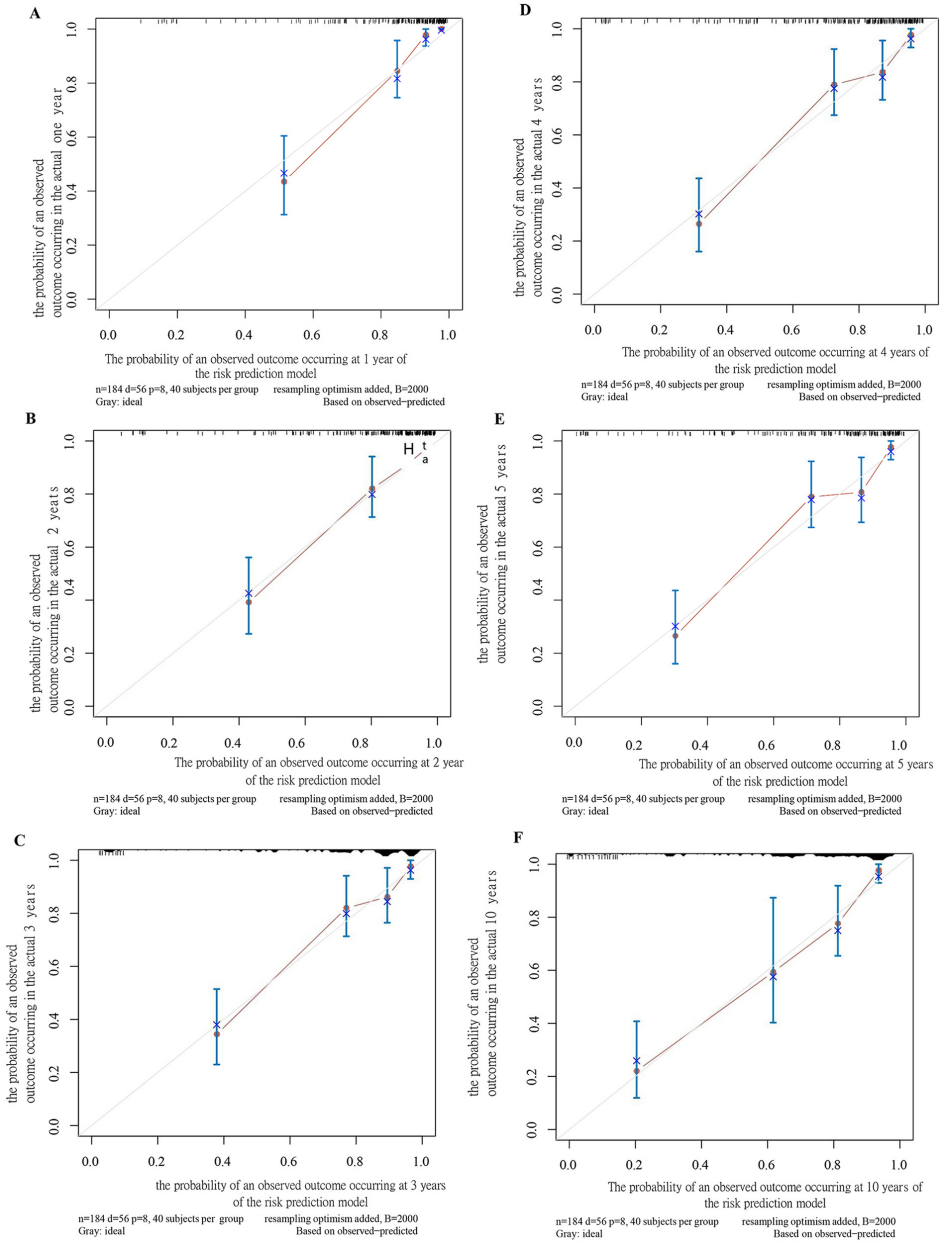
Calibration plot of the SCD prediction model for children at 1 year **(A)**, 2 years **(B)**, 3 years **(C)**, 4 years **(D)**, 5 years **(E)**, and 10 years **(F)**.

A nomogram was developed to visualize the predictive model (Figure 1). Among all features, an LVPWd of 55 mm contributed the highest score of 100 points, whereas age <1 year, female sex, positive family history, pathological Q waves, fQRS, and arrhythmias contributed 47, 25, 30, 28, 30, and 21 points, respectively (Supplementary Table S2). The results indicated that a higher total score was associated not only with an increased risk at a given time point but also with a higher risk over a prolonged period. For instance, a score of 93 corresponded to a 10% risk at 1 year and a 20%–30% risk over a 10-year follow-up period (Supplementary Table S3). The predicted probabilities of the model were consistent with the observed probabilities (Figure 2).

## Discussion

This study proposed a nomogram-based model for predicting SCD-related events in children with HCM. The model incorporated multimodal features and demonstrated high performance in both training and testing datasets. Moreover, it provided reliable outcome predictions for time frames ranging from 1 to 10 years, with high AUC values.

Prognostic prediction of HCM in children is particularly challenging due to its complex etiology, diverse clinical phenotypes, and significant population heterogeneity. Furthermore, fewer large-scale, multicenter follow-up studies have been conducted in children compared to adults. A retrospective cohort study from the United Kingdom involving 687 children (aged 0–16 years) with HCM reported that prognosis was influenced by age and etiology (11, 16). The study revealed that HCM diagnosed before 1 year of age or resulting from congenital metabolic disorders was associated with a poor prognosis, with 5-year survival rates of 80.5% and 66.4%, respectively. A European multicenter cohort study of 301 infants with HCM found a 5-year survival rate of 85% (17) and identified inherited metabolic diseases, cardiac symptoms, and impaired LV systolic function as predictors of all-cause mortality. Similarly, a study by Alashi et al. demonstrated that the presence of symptoms, ventricular tachycardia, and a higher *z*-score for interventricular septal thickness were independently associated with adverse composite events (18). A single-center study in Egypt reported 51 deaths among 128 patients, including 36 presenting before 1 year of age (19), primarily due to limited medical resources. Among these patients, only eight underwent surgery. Severe LVH, sinus tachycardia, and supraventricular tachycardia were identified as independent risk factors for mortality. However, none of these studies included Asian populations. Consistent with these findings, the present study confirmed that age <1 year was an independent risk factor for SCD-related events in children with HCM, underscoring the importance of early identification and timely intervention for this patient group.

Sex was identified as another important prognostic factor. A cohort study by Abou et al., which included 1,010 pediatric patients with HCM, demonstrated that females had a higher likelihood of developing composite outcomes (20). Similarly, a larger study involving 5,873 patients reported that, irrespective of genotype, females exhibited a higher risk of mortality and more severe heart failure symptoms (21), another study performed by Niccolo et al. also revealed that female was a risk factor for long-term outcomes in obstructive HCM population who underwent septal reduction therapy (22). Although not all studies focused on pediatric populations, the overall trend aligns with the present findings, indicating that female patients with HCM tend to have worse prognoses. However, the underlying mechanism still required further analysis. A previous study showed that a higher penetrance of *MYBPC3* mutation carriers in male subjects than in female subjects, and the high penetrance caused by the mutation allows male subjects to exhibit the disease earlier and receive earlier treatment (23). The anatomical structure difference between male and female may also influence the severity of HCM, Kim reported that smaller cardiac chambers could be associated with higher frequency and severity of LVOT obstruction in females (24).

ECG abnormalities are critical indicators of disease prognosis and often precede clinical symptoms. Most HCM patients exhibit ECG changes, including myocardial depolarization and repolarization abnormalities and arrhythmias, primarily due to myocardial fibrosis, as confirmed by contrast-enhanced CMR (25). Previous studies have proposed that specific ECG abnormalities, such as fQRS, are strongly associated with delayed conduction caused by myocardial scarring and can predict the prognosis of various cardiac diseases. Jeffrey et al. identified fQRS as an independent risk factor for mortality and SCD in heart failure patients (26). Ratheendran and colleagues linked fQRS to LGE signals in 36 HCM patients, highlighting its role as an indirect marker of fibrosis (27). In this study, multivariate analysis revealed that fQRS and other ECG abnormalities, including pathological Q waves and arrhythmias, were independent prognostic factors for HCM in children. However, due to the small sample size, different types of arrhythmias were combined for analysis, and stratification was not performed, which may have introduced bias.

LVH is the hallmark of HCM, and severe LVH has been associated with more extensive myocardial fibrosis and a higher risk of SCD (28). Several large pediatric studies have validated the importance of severe LVH in risk stratification for children with HCM (6, 13). In this study, increased IVSd and LVPWd were identified as independent risk factors for adverse outcomes. However, the lack of standardized measurement criteria, such as *z*-scores to adjust for age and body surface area, may have introduced confounding factors. Future studies utilizing adjusted LV parameters are warranted to provide more robust findings. We also noticed that no significant difference was found in LVDd between two groups. However, the patients in SCD group showed a predominance of enlarged cardiac silhouette, these could be partially explained by the multi-facet influencing factors of cardiac silhouette undert x-ray examination. Sasamoto reported that pericardial effusion could lead to enlarged cardiac silhouette (29), while Avner showed patients' position could also lead to different heart size (30). Therefore, the LVDd derived from echocardiography was more accurate and used as reference in this study.

In the present study, a novel prediction model was constructed based on the results of multivariate analysis to predict the AUC for the outcome of interest in children with HCM at 1, 2, 3, 4, 5, and 10 years. The AUC values consistently exceeded 0.8, demonstrating high predictive accuracy. Internal validation was conducted using bootstrap sampling repeated 2,000 times. Compared with the aforementioned models (HCM-Risk-Kids and PRIMaCY), our proposed model included both primary HCM and secondary HCM, covering a wider range of research subjects. Although the two previous research models have a large research span, the long time span also leads to possible changes in examination methods among different periods. Additionally, data obtained from multiple medical centers may have different measurements criteria, which can affect the standardization of parameters and model performance. We also noticed that some echographic data were missing in the previously mentioned two models, although CMR was not completed for all participants in this study, the echocardiography was performed in every child, which made our model more complete and reliable. Moreover, the patient cohort in this study was composed exclusively of Chinese children, which may render the model particularly relevant for evaluating SCD-related events in future studies focusing on Chinese populations.

This study, however, has several limitations. First, it was a single-center, retrospective study with a relatively small sample size. Thus, validation through large, multicenter, prospective studies involving the Chinese paediatric HCM population is necessary. Second, some follow-up data were obtained through parental recall, which may have introduced recall bias. Young children were often unable to articulate their symptoms, resulting in most clinical manifestations being reported by their parents. Finally, the model has not undergone external validation. Future studies should include additional cases to enable comprehensive validation.

## Conclusions

Age, sex, family history, pathological *Q* waves, fQRS, combined arrhythmias, interventricular septal thickness, and LV posterior wall thickness are prognostic factors for HCM in children. This study presents an internally validated predictive model for SCD in pediatric HCM patients. External validation is necessary before its clinical implementation.

## Data Availability

The original contributions presented in the study are included in the article/Supplementary Material, further inquiries can be directed to the corresponding author.

## Supplementary material

Supplementary Figure 1 Area under the Receiver Operating Characteristic (ROC) Curve (AUC) of the Risk Prediction Model Over Time for Predicting the Development of SCD in Children.

Supplementary Figure 2 ROC Curves for Predicting SCD in Children at 1 Year (A), 2 Years (B), 3 Years (C), 4 Years (D), 5 Years (E), and 10 Years (F).
