# Supplementary figures and images for: Developing a risk prediction model for sudden cardiac death in children with hypertrophic cardiomyopathy
Source: Front Pediatr. 2025 Aug 14;13:1628585. doi: 10.3389/fped.2025.1628585 (PMC12391110; doi:10.3389/fped.2025.1628585)

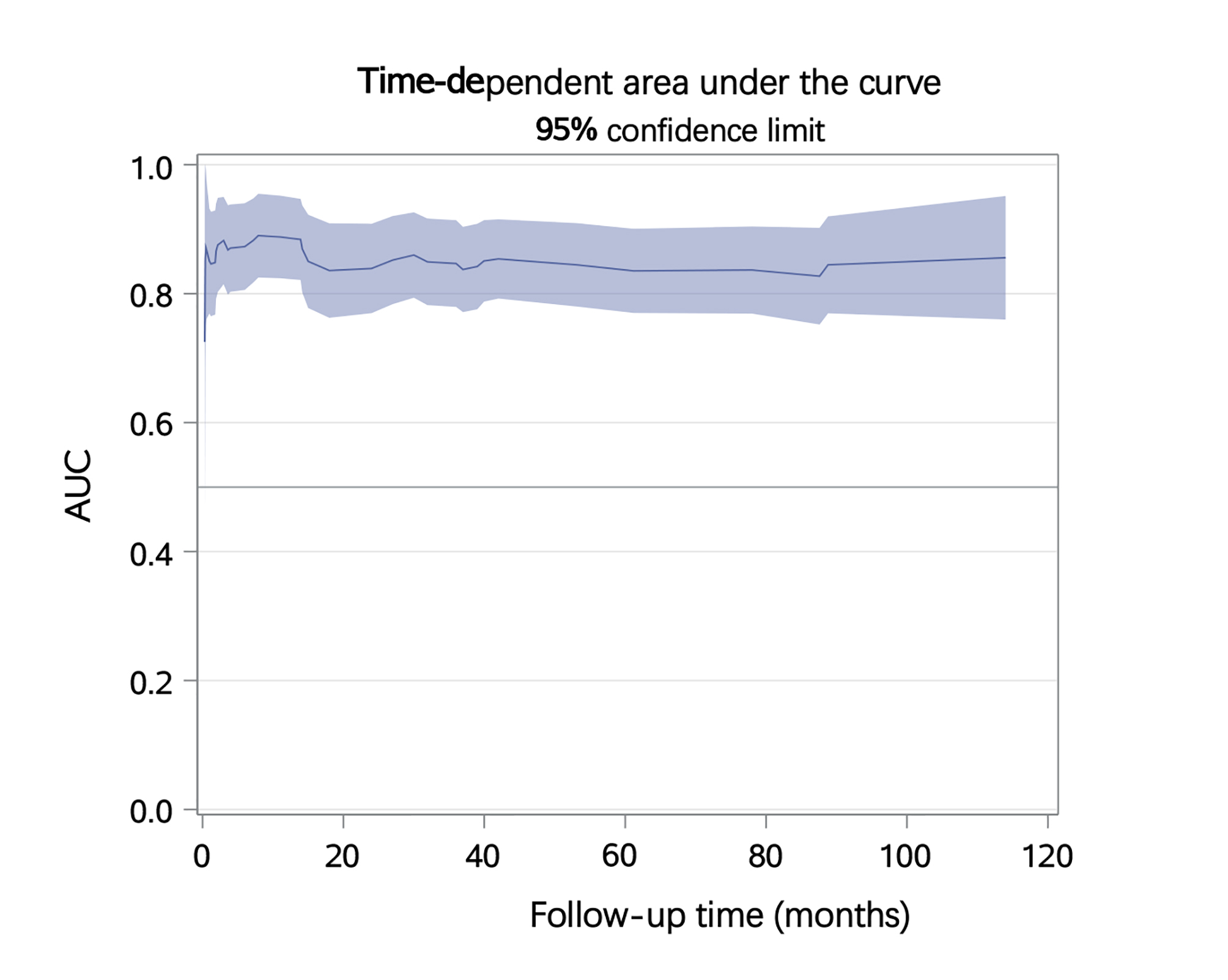

Supplement: Supplementary Figure 1 — Area under the Receiver Operating Characteristic (ROC) Curve (AUC) of the Risk Prediction Model Over Time for Predicting the Development of SCD in Children. [file Image1.jpeg]

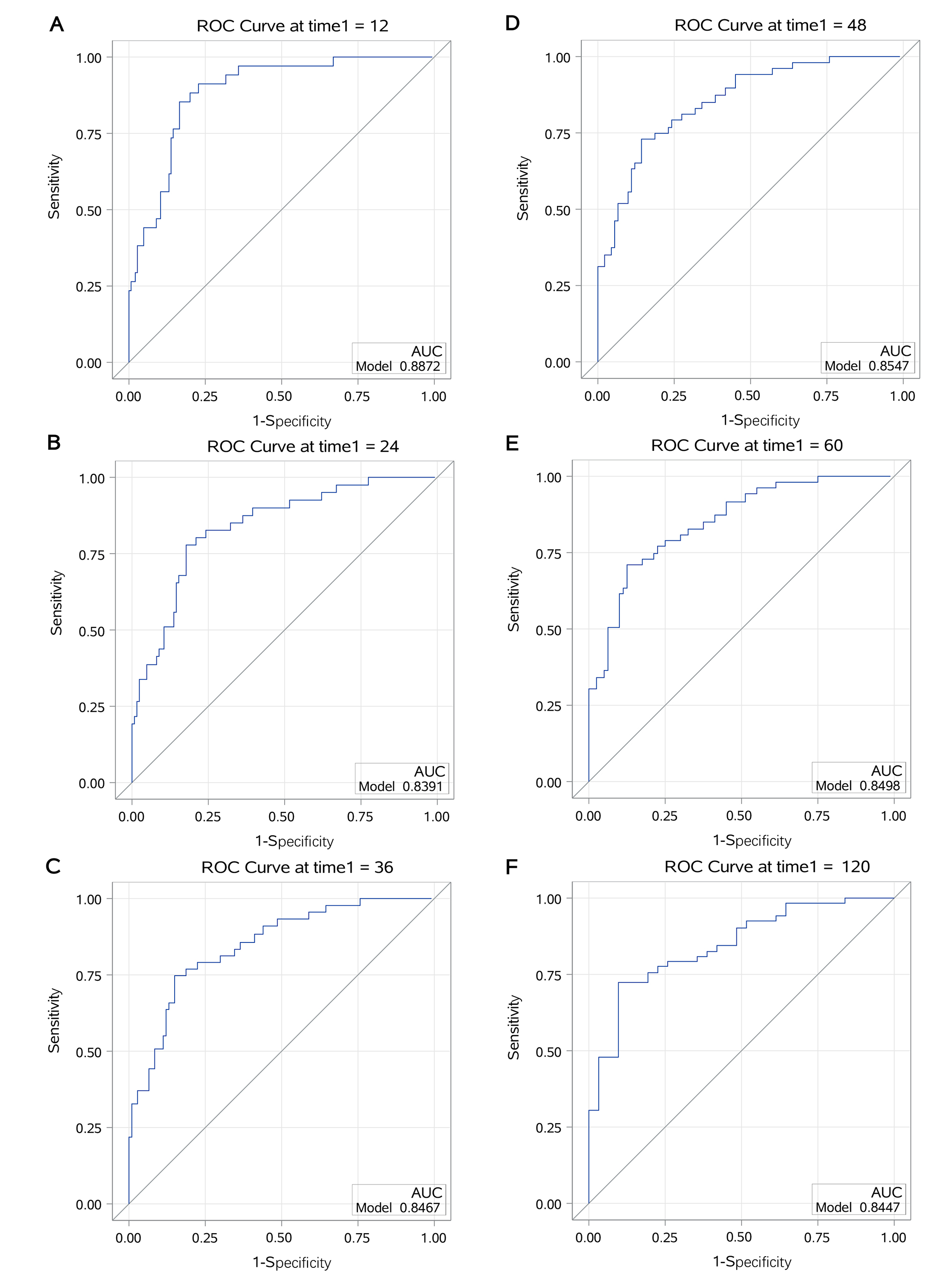

Supplement: Supplementary Figure 2 — ROC Curves for Predicting SCD in Children at 1 Year (A), 2 Years (B), 3 Years (C), 4 Years (D), 5 Years (E), and 10 Years (F). [file Image2.jpeg]
