# Supplementary material for: Developing a risk prediction model for sudden cardiac death in children with hypertrophic cardiomyopathy
Source: Front Pediatr. 2025 Aug 14;13:1628585. doi: 10.3389/fped.2025.1628585 (PMC12391110; doi:10.3389/fped.2025.1628585)
Supplement: Supplementary file 3 [file Datasheet1.docx]

**Supplementary Table 1.** Comparison of baseline characteristics between groups

|  | **None SCD group**  **(n=128)** | **SCD group**  **(n=56)** | **P** |
| --- | --- | --- | --- |
| **Age** |  |  |  |
| <1 year | 37 (28.91) | 25 (44.64) | 0.038 |
| ≥1 year | 91 (71.09) | 31 (55.36) |  |
| **Sex** |  |  |  |
| Female | 41 (32.03) | 28 (50.00) | 0.021 |
| Male | 87 (67.97) | 28 (50.00) |  |
| **Etiology** |  |  |  |
| Secondary | 22 (17.19) | 21 (37.50) | 0.003 |
| Primary | 106 (82.81) | 35 (62.50) |  |
| **Family History** | 26 (20.31) | 23 (41.07) | 0.003 |
| **Initial Symptoms** |  |  |  |
| Chest pain/tightness | 30 (23.44) | 8 (14.29) | 0.158 |
| Fatigue/decreased exercise tolerance | 53 (41.41) | 34 (60.71) | 0.016 |
| Palpitations | 10 (7.81) | 2 (3.57) | 0.351 |
| Syncope | 11 (8.59) | 12 (21.43) | 0.015 |
| Cardiogenic shock | 6 (4.69) | 8 (14.29) | 0.034 |
| Atypical presentation | 7 (5.47) | 6 (10.71) | 0.220 |
| **Initial Signs** |  |  |  |
| Murmur | 46 (35.94) | 19 (33.93) | 0.793 |
| Gallop rhythm | 2 (1.56) | 1 (1.79) | >0.999 |
| **Initial Heart Failure Classification** |  |  |  |
| Class III–IV | 25 (19.53) | 23 (41.07) | 0.002 |
| **Chest X-ray** |  |  |  |
| Enlarged cardiac silhouette | 48 (37.50) | 39 (69.64) | <0.001 |
| Pulmonary congestion | 2 (1.56) | 3 (5.36) | 0.166 |
| **Electrocardiogram (ECG)** |  |  |  |
| ST-T changes | 101 (78.91) | 52 (92.86) | 0.020 |
| Pathological Q waves | 32 (25.00) | 32 (57.14) | <0.001 |
| Fragmented QRS | 25 (19.53) | 26 (46.43) | <0.001 |
| Left ventricular hypertrophy | 14 (10.94) | 11 (19.64) | 0.113 |
| NSVT (nonsustained ventricular tachycardia) | 27 (21.09) | 18 (32.14) | 0.109 |
| Supraventricular tachycardia (SVT) | 33 (25.78) | 23 (41.07) | 0.038 |
| Atrioventricular (AV) block | 25 (19.53) | 14 (25.00) | 0.404 |
| Wolff-Parkinson-White (WPW) syndrome | 10 (7.81) | 13 (23.21) | 0.004 |
| Arrhythmia | 64 (50.00) | 42 (75.00) | 0.002 |
| **Echocardiography** |  |  |  |
| LVDd, (mm) | 35.31 ± 9.10 | 34.98 ± 9.42 | 0.823 |
| LVDs, (mm) | 25.89 ± 8.23 | 24.89 ± 9.17 | 0.467 |
| IVSd, (mm) | 8.3 (6, 13.8) | 10.7 (8.05, 17.55) | 0.026 |
| LVPWd, (mm) | 6.0 (4.75, 7.6) | 8.7 (5.5, 11.8) | <0.001 |
| IVSd / LVPWd | 1.27 (1, 2.13) | 1.18 (0.98, 1.6) | 0.424 |
| LVEF, (%) | 71 (66, 77.5) | 68 (60.5, 73.5) | 0.009 |
| LVOTO | 26 (20.31) | 10 (17.86) | 0.699 |
| **CMR#** |  |  |  |
| **LGE positive** | 15 (22.73) | 16 (55.17) | 0.002 |

Values are n (%) or mean ± standard deviation. CMR: cardiovascular magnetic resonance; LVDd: LV end-diastolic diameter; LVDs: LV end-systolic diameter; IVSd: interventricular septum diastolic thickness; LVPWd: LV posterior wall diastolic thickness; LVEF: LV ejection fraction; LVOTO: LV outflow tract obstruction; LGE: late gadolinium enhancement.

#: A total of 95 people underwent CMR examination; /: Fisher's exact test for no statistics

**Supplementary Table 2.** Scoring Assignment for Predictors in the Nomogram Model.

| Variables | Score |
| --- | --- |
| **Age** |  |
| <1 year | 47 |
| ≥1 year | 0 |
| **Sex** |  |
| Female | 25 |
| Male | 0 |
| **Family History** |  |
| No | 0 |
| Yes | 30 |
| **Pathological Q waves** |  |
| No | 0 |
| Yes | 28 |
| **fQRS** |  |
| No | 0 |
| Yes | 30 |
| **Arrhythmia** |  |
| No | 0 |
| Yes | 21 |
| **IVSd (mm)** |  |
| 0 | 0 |
| 5 | 7 |
| 10 | 15 |
| 15 | 22 |
| 20 | 30 |
| 25 | 37 |
| 30 | 45 |
| 35 | 52 |
| **LVPWd (mm)** |  |
| 0 | 0 |
| 5 | 9 |
| 10 | 18 |
| 15 | 27 |
| 20 | 36 |
| 25 | 45 |
| 30 | 55 |
| 35 | 64 |
| 40 | 73 |
| 45 | 82 |
| 50 | 91 |
| 55 | 100 |

IVSd: interventricular septum diastolic thickness; LVPWd: LV posterior wall diastolic thickness.

**Supplementary Table 3.** Summary Scores of the Nomogram Model and the Probability of Observing Outcomes at 1, 2, 3, 4, 5, and 10 Years.

| Total Score | Probability of observed outcome at 1 year | Total Score | Probability of observed outcome at 2 years | Total Score | Probability of observed outcome at 3 years | Total Score | Probability of observed outcome at 4 years | Total Score | Probability of observed outcome at 5 years | Total Score | Probability of observed outcome at 10 years |
| --- | --- | --- | --- | --- | --- | --- | --- | --- | --- | --- | --- |
| 93 | 0.1 | 84 | 0.1 | 79 | 0.1 | 73 | 0.1 | 72 | 0.1 | 61 | 0.1 |
| 114 | 0.2 | 106 | 0.2 | 101 | 0.2 | 95 | 0.2 | 93 | 0.2 | 83 | 0.2 |
| 128 | 0.3 | 120 | 0.3 | 115 | 0.3 | 108 | 0.3 | 107 | 0.3 | 96 | 0.3 |
| 138 | 0.4 | 130 | 0.4 | 125 | 0.4 | 119 | 0.4 | 117 | 0.4 | 107 | 0.4 |
| 147 | 0.5 | 139 | 0.5 | 134 | 0.5 | 128 | 0.5 | 126 | 0.5 | 116 | 0.5 |
| 155 | 0.6 | 147 | 0.6 | 142 | 0.6 | 136 | 0.6 | 134 | 0.6 | 124 | 0.6 |
| 163 | 0.7 | 155 | 0.7 | 150 | 0.7 | 144 | 0.7 | 142 | 0.7 | 132 | 0.7 |
| 172 | 0.8 | 163 | 0.8 | 158 | 0.8 | 152 | 0.8 | 151 | 0.8 | 140 | 0.8 |
| 182 | 0.9 | 174 | 0.9 | 169 | 0.9 | 163 | 0.9 | 161 | 0.9 | 151 | 0.9 |
